# Vitamin D status and COVID-19 severity

**DOI:** 10.4102/sajid.v37i1.359

**Published:** 2022-04-26

**Authors:** Senrina Kalichuran, Sarah A. van Blydenstein, Michelle Venter, Shahed Omar

**Affiliations:** 1 Department of Internal Medicine, Faculty of Health Sciences, School of Clinical Medicine, University of the Witwatersrand, Johannesburg, South Africa; 2 Department of Pulmonology, Faculty of Internal Medicine, Chris Hani Baragwanath Academic Hospital, Johannesburg, South Africa; 3 Department of Infectious Diseases, Chris Hani Baragwanath Academic Hospital, Johannesburg, South Africa; 4 Department of Critical Care, Faculty of Health Sciences, University of the Witwatersrand, Johannesburg, South Africa; 5 Department of Critical Care, Chris Hani Baragwanath Academic Hospital, Johannesburg, South Africa

**Keywords:** vitamin D, COVID-19; severity, Johannesburg, South Africa

## Abstract

**Background:**

Age, body mass index (BMI) and pre-existing comorbidities are known risk factors of severe coronavirus disease 2019 (COVID-19). In this study we explore the relationship between vitamin D status and COVID-19 severity.

**Methods:**

We conducted a prospective, cross-sectional descriptive study. We enrolled 100 COVID-19 positive patients admitted to a tertiary level hospital in Johannesburg, South Africa. Fifty had symptomatic disease (COVID-19 pneumonia) and 50 who were asymptomatic (incidental diagnosis). Following written informed consent, patients were interviewed regarding age, gender and sunlight exposure during the past week, disease severity, BMI, calcium, albumin, magnesium and alkaline phosphatase levels. Finally, blood was collected for vitamin D measurement.

**Results:**

We found an 82% prevalence rate of vitamin D deficiency or insufficiency among COVID-19 patients. Vitamin D levels were lower in the symptomatic group (18.1 ng/mL ± 8.1 ng/mL) than the asymptomatic group (25.9 ng/mL ± 7.1 ng/mL) with a *p*-value of 0.000. The relative risk of symptomatic COVID-19 was 2.5-fold higher among vitamin D deficient patients than vitamin D non-deficient patients (confidence interval [CI]: 1.14–3.26). Additional predictors of symptomatic disease were older age, hypocalcaemia and hypoalbuminaemia. Using multiple regression, the only independent predictors of COVID-19 severity were age and vitamin D levels. The patients exposed to less sunlight had a 2.39-fold increased risk for symptomatic disease compared to those with more sunlight exposure (CI: 1.32–4.33).

**Conclusion:**

We found a high prevalence of vitamin D deficiency and insufficiency among patients admitted to hospital with COVID-19 and an increased risk for symptomatic disease in vitamin D deficient patients.

## Introduction

The world is currently battling the coronavirus disease 2019 (COVID-19) pandemic, caused by severe acute respiratory syndrome coronavirus 2 (SARS-Co-V2). This coronavirus was first identified in Wuhan, China in December 2019.^1^ This viral infection mainly affects the respiratory system with a range of immune-mediated disease severity. While some patients are asymptomatic, others present with hypoxia and even multiorgan dysfunction. To date, several vaccines have been developed, but there has been a significant delay in vaccination across the African continent.^2^ Risk factors that predict severe disease include age, body mass index (BMI) and pre-existing comorbidities. In addition, the role of vitamin D deficiency has also been explored as a risk factor for severity.^3^

Vitamin D3 (cholecalciferol) is an important secosteroid that is converted from previtamin D in the skin by exposure to ultraviolet B (UVB) rays from the sun, which breaks a carbon to carbon bond and this process is then followed by thermal isomerisation.^4^ It is further hydroxylated in the liver to 25-hydroxyvitamin D (25[OH]D) and then to its active form 1,25-dihydroxyvitamin D (1,25[OH]_2_D) mainly in the kidney as well as in other tissues including cells of the respiratory tract and most cells of the immune system.^5^ Vitamin D is naturally occurring in fatty fish at moderate levels and may be supplemented as oral preparations or by the fortification of dairy products or cereals.^6^

Vitamin D deficiency is common globally.^7^ While many chronic illnesses predispose patients to this problem, reduced sunlight exposure is a major cause of vitamin D deficiency even in countries with higher levels of sunlight.^8,9^ Deficiency is brought about by seasonal changes, darker skin pigmentation, cultural dressing norms and indoor lifestyle or occupation.^5,7,8,10,11^

Cytochrome p450 27B1 or 1-alpha-hydroxylase (CYP27B1) is the enzyme responsible for conversion of 25(OH)D to 1,25(OH)_2_D. Vitamin D receptors (VDR) and CYP27B1that are found in respiratory tissue cells and most cells of the immune system allow for larger concentrations of 1,25(OH)_2_D due to lack of feedback mechanisms as are found in the renal conversion process.^6^ In other respiratory viruses, such as respiratory syncytial virus and influenza, vitamin D has a role in preventing disease as well as limiting disease severity.^12,13^ It has also been found to attenuate the cytokine storm associated with COVID-19 disease which results in multiorgan dysfunction and acute respiratory distress syndrome^6^; this exaggerated immune response is dampened by adequate levels of vitamin D, which shifts T-cells from a proinflammatory to a tolerogenic state by inhibiting proinflammatory cytokines such as interferon γ, tumour necrosis factor α and interleukins 2, 9 and 22.^6^

Currently in South Africa, there is a paucity of data regarding vitamin D deficiency prevalence and particularly within the population of patients with COVID-19 infection. This study aims to address the knowledge gap in vitamin D status and COVID-19 severity in a South African population.

## Methods

### Study setting

This study was conducted at Chris Hani Baragwanath Academic Hospital, a tertiary level hospital in Johannesburg, South Africa. It is located at a latitude of 26.2 ˚S of the equator. All patients enrolled in the study were COVID-19 positive and were admitted to the adult COVID-19 wards.

### Study design

This was a prospective, cross-sectional descriptive study. Participants were COVID-19 positive (confirmed by reverse transcription polymerase chain reaction [RT-PCR]) who were admitted to Chris Hani Baragwanath Academic Hospital in the COVID-19 wards between September 2020 and February 2021.

### Participants

Consecutive sampling was used to enrol 100 participants during the study period. Of these patients, 50 were admitted in the surgical COVID-19 wards with an incidental diagnosis of COVID-19 (asymptomatic) and 50 were admitted in the medical wards with COVID-19 pneumonia (symptomatic). All patients were confirmed to have SARS-CoV-2 infection during the current admission by a positive RT-PCR result on nasopharyngeal swabs. Those excluded were patients with active pulmonary tuberculosis, active malignancy, gastrointestinal malabsorption, pancreatic insufficiency, chronic kidney disease, parathyroid disease as well as patients who were currently pregnant or already on vitamin D supplementation.

### Data collection

Eligible patients or a suitable proxy, in the case of hypoxia or confusion, were informed of the study and were invited to participate. Those patients who agreed to participate were asked to sign a consent form. Each participant was allocated a randomised study number to anonymise their data on the questionnaire form. To complete the questionnaire, patients were then interviewed regarding their age, gender, and sunlight exposure within the past week. Each patient was weighed and their height measured to calculate their BMI. Information collected from each file was the severity of COVID-19 disease as well as calcium, albumin, magnesium and alkaline phosphatase levels which form part of the admission blood workup for every COVID-19 patient. A 3 mL – 5 mL cuffed venous sample of blood was taken from each patient in an acid citrate dextrose tube. The samples were transported under cold chain conditions to the laboratory for vitamin D measurement.

### Vitamin D testing and definitions

Vitamin D levels were measured using a double sandwich immunoassay using a chemiluminescent label at a South African National Accreditation System (ISO 15189) accredited laboratory. The instrument used was the Abbott © Architect. This method is traceable to the reference method, namely liquid chromatography mass spectrometry (LC-MS) and meets the required standards for clinical testing.^14^ There is currently no consensus on the definition of vitamin D deficiency, insufficiency and adequacy.^7^ For the purposes of this study, we have chosen the Endocrine Society guidelines.^15^ These definitions are outlined in Table 1.

**TABLE 1:** The prevalence of Vitamin D deficiency and Vitamin D insufficiency.

Vitamin D status	Prevalence	CI	*n*
**Vitamin D deficiency (< 20 ng/mL)**			
All COVID-19 patients	0.44	0.39–0.49	100
Symptomatic COVID-19	0.66	0.59–0.73	50
Asymptomatic COVID-19	0.22	0.16–0.28	50
**Vitamin D insufficiency (21 ng/mL – 29 ng/mL)**			
All COVID-19 patients	0.38	0.33–0.43	100
Symptomatic COVID-19	0.26	0.2–0.32	50
Asymptomatic COVID-19	0.50	0.43–0.57	50
**Adequate Vitamin D levels (> 30 ng/mL)**			
All COVID-19 patients	0.18	0.14–0.22	100
Symptomatic COVID-19	0.08	0.04–0.12	50
Asymptomatic COVID-19	0.28	0.22–0.34	50

CI, confidence interval; COVID-19, coronavirus disease 2019.

### Statistical analysis

All data obtained were entered onto a spreadsheet on Microsoft Excel and then entered onto Statistica version 13.3 (StatSoft, United States [US]). The distribution of data was determined from histograms and Lilliefor’s test. Categorical variables were presented as counts (*n*) and percentages and comparisons were made in symptomatic and asymptomatic groups using the Chi-square test. Continuous variables were summarised as means with standard deviations (s.d.) for normally distributed data and medians with interquartile ranges for non-standard distributed data. Independent variables were compared in symptomatic versus asymptomatic groups using Mann–Whitney U test for independent medians and Student’s *T*-test for independent means. Using the results from the univariate analysis, five variables with a *p*-value ≤ 0.05 were used in a multivariate model to predict COVID-19 severity. A *p*-value of less than 0.05 was considered statistically significant.

### Sample size

Using a 5% precision and 80% confidence level, it was determined that we would require 81 patients to detect a 50% increase in vitamin D deficiency in symptomatic patients versus asymptomatic patients. This model is based on a 50% increase in the prevalence of vitamin D deficiency from a baseline of 28%, which is the highest prevalence in a South African population.^8^

### Study objectives

The primary objective of this study is to determine the prevalence of vitamin D insufficiency and deficiency in patients with asymptomatic and symptomatic COVID-19 disease at Chris Hani Baragwanath Academic Hospital.

Secondary objectives are to compare the vitamin D levels in the two groups of COVID-19 severity, determine the risk of symptomatic disease and vitamin D deficiency, determine the relationship between COVID-19 severity and sunlight exposure and to compare the following between symptomatic and asymptomatic groups: age, gender, BMI, calcium, magnesium, alkaline phosphatase and albumin levels.

### Ethical considerations

Ethics approval was obtained from the Human Research Ethics Committee (Medical) of the University of the Witwatersrand (ethics approval number M200653). Registration with the National Health Research Database has been completed (GP_202109_063) and approval is pending.

## Results

### Primary objective

#### Vitamin D levels and COVID-19 severity

We found an 82% prevalence rate of vitamin D deficiency or insufficiency among COVID-19 patients. The mean vitamin D levels for deficiency, insufficiency and adequacy were 14.6 ng/mL (s.d. ±4.3), 24.3 ng/mL (s.d. ±2.8) and 35.3 ng/mL (s.d. ±3.9), respectively. Table 1 shows the prevalence of vitamin D deficiency, insufficiency and adequacy among all patients, symptomatic patients and asymptomatic patients.

### Secondary objectives

#### Vitamin D levels and other predictors of severity

Vitamin D levels and predictors of severity of illness are given in Table 2. Significant differences are noted between the symptomatic and asymptomatic groups in terms of age, BMI, vitamin D, calcium and albumin levels. There was no significant difference found between the two groups in magnesium and alkaline phosphatase levels.

**TABLE 2:** Differences between symptomatic and asymptomatic COVID-19.

Variable	All (*n* = 100)	Asymptomatic COVID-19 (*n* = 50)	Symptomatic COVID-19 (*n* = 50)	*p*
**Age** (years)				0.000*
Mean	45.7 ±15.9	39.3 ±14.9	52.2 ±14.2	
**Gender** (%)				
Male	59	29	30	
Female	41	21	20	
**BMI** (kg/m^2^), median				0.05
Mean	25.7	24.7	27.2	
**Vitamin D** (ng/mL), mean				0.000*
Mean	22 ±8.4	25.9 ±7.1	18.1 ±8.1	
**Calcium** (mmol/l), mean				0.002*
Mean	2.3 ±0.1	2.3 ±1.2	2.2 ±0.2	
**Albumin** (g/L), mean				0.003*
Mean	36 ±6.1	38 ±5.9	34 ±5.8	
**Magnesium**				0.71
(mmol/L)	0.8	0.8	0.9	
**Alkaline Phosphatase**				0.37
(U/L)	89	92	84	

BMI, body mass index; COVID-19, coronavirus disease 2019.

* , Significance < 0.05.

#### Risk of symptomatic COVID-19

The relative risk (RR) of symptomatic COVID-19 was 2.5 (1.66–3.69) times higher among vitamin D deficient patients when compared to vitamin D non-deficient patients.

#### Multiple regression model

Using variables from the univariate analysis in Table 2, we selected those variables that had a *p*-value of ≤ 0.05 and created a multiple regression model to determine the independent predictors of severity of COVID-19. This is shown in Table 3. Only age and vitamin D levels were found to be independent predictors of severity.

**TABLE 3:** COVID-19 severity regression model.

Variable	*b* *	Standard error of b*	*p*
Intercept: 0.42			
Age	−0.26	0.09	0.004*
BMI	−0.07	0.09	0.41
Calcium	0.13	0.10	0.18
Albumin	0.15	0.10	0.13
25(OH)_2_D	0.30	0.09	0.001*

*R* = 0.58, *R*² = 0.34, Adjusted *R*² = 0.31, *F*(5,94) = 9.77, *p* < 0.000, *n* = 100.

BMI, body mass index.

* , Significance < 0.05.

#### Relationship between severity of illness and sunlight

Table 4 compares the severity of illness with sunlight exposure. The RR of symptomatic disease if the patient was exposed to less than 6 h of sunlight per week was 2.39-fold higher (CI: 1.32–4.33) when compared to at least 1–2 h per day of sunlight exposure.

**TABLE 4:** Sunlight exposure and ethnicity in disease severity.

Variable	All *n* = 100	Asymptomatic (*n* = 50)		Symptomatic (*n* = 50)		*X* ^2^	*p*
		*n*	%	*n*	%		
**Sunlight**						7.29	0.007*
Less sunlight < 6 h/week	79	34	43	45	57		
More sunlight > 6 h/week	21	16	76.2	5	23.8		

* , Significance < 0.05.

## Discussion

The main finding of our study is that 82% of the patients with COVID-19 infection had insufficient or deficient vitamin D levels (< 30 ng/mL). More specifically, 38% of our cohort were insufficient (21–29 ng/mL) and an additional 44% were deficient (< 20 ng/mL).

Using the same definition of vitamin D deficiency and insufficiency as our study, a study performed in India found similar results to our study with a combined prevalence of vitamin D deficiency (< 20 ng/mL) and insufficiency (20 ng/mL – 30 ng/mL) at 89.1%.^16^ This comprised 58.9% of vitamin D deficient patients and 30.2% with vitamin D insufficiency. Likewise, an Algerian study found a prevalence of vitamin D insufficiency (20 ng/mL – 30 ng/mL) and deficiency (< 20 ng/mL) of 75.1%. Of this group, 55.9% of patients were vitamin D deficient.^17^

Radujkovic et al. found a prevalence of vitamin D insufficiency (12 ng/mL – 20 ng/mL) and deficiency (< 12 ng/mL) of 66% and 22%, respectively.^18^ Panagiotou et al. found a high prevalence of combined vitamin D deficiency and insufficiency of 66.4% (< 20 ng/mL).^19^ Campi et al. found that 64% of patients had vitamin D deficiency or insufficiency (< 30 ng/mL).^20^ A recent Belgian study also found a high prevalence of vitamin D deficiency (< 20 ng/mL) of close to 60%.^21^ This is significantly higher than the 44% vitamin D deficiency found in our study. Key differences from our study include differing definitions of vitamin D status, an older population and higher latitude. These differences may well explain the varied prevalence rates of vitamin D deficiency and insufficiency compared to our study population.

Another important finding of our study was that median vitamin D levels significantly differed between the symptomatic and asymptomatic groups at 18.1 ng/mL and 25.9 ng/mL, respectively. Similar findings of differences in vitamin D levels between populations with severe COVID-19 disease versus those with milder disease have previously been noted. Lower vitamin D levels were found in patients with severe disease, with median levels of 14.6 ng/mL compared to 18.6 ng/mL in patients with less severe disease. This relationship with severity was borne out by increasing oxygen requirements.^18^

Retrospective data also indicated lower vitamin D levels among intensive care unit (ICU) patients compared to non-ICU patients at 13.42 ng/mL and 19.27 ng/mL, respectively.^19^ Decreasing mean vitamin D levels were found among outpatients (30.8 ng/mL), hospitalised non-ICU patients (22.4 ng/mL) and ICU patients (14.4 ng/mL).^20^ De Smet et al. found decreasing median vitamin D levels in patients with increasing severity of pneumonia as described by chest CT scan.^21^ Bennouar et al. found lower mean vitamin D levels in non-survivors (14.1 ng/mL) compared to survivors (23.9 ng/mL).^17^

Despite differing vitamin D levels, the overall finding of lower vitamin D levels among more severely ill patients is consistent with our study findings. Vitamin D levels in our study are higher than some of those discussed above. This may be explained by differences in age and latitude. We did not calculate the severity of illness in our study group and this cannot be discounted as a contributor to the differences above.

Additional predictors of symptomatic disease identified in our study are older age, hypocalcaemia and hypoalbuminaemia. There was also a trend to greater severity in patients with a higher BMI. Data from other studies show a consistent relationship between older age and more severe disease.^16,20,22,24,25,26^ Our data showed a median age of 52 years in the symptomatic group versus 39 years in the asymptomatic group. Higher BMI has been identified as a risk factor for severe disease or poor outcomes such as the need for invasive ventilation or death.^16,20,22,25,26^ Hypocalcaemia is a significant finding in severe COVID-19 disease;^17,27^ however, the underlying relationship with vitamin D needs further investigation. Being a negative acute phase reactant, it was not surprising that albumin was found to be lower in patients with more severe COVID-19 disease.^17,22,28^

A large proportion (79%) of the patients included in our study were exposed to less than 6 h of sunlight per week. Interestingly, we found that these patients were at a greater than 2-fold increased risk for symptomatic disease compared to those with more than 6 h of sunlight exposure per week. Whittemore found a significant correlation between proximity to the equator and COVID-19 related mortality.^29^ He suggested a relationship between sunlight exposure and COVID-19 mortality; however, this study fails to adjust for the lower average population age in regions closer to the equator. Such an adjustment was considered by Rhodes et al. who found similar correlations between latitude and COVID-19 related mortality.^30^ Guasp et al. noted a lower incidence of COVID-19 infections in countries exposed to more sunlight than those with less sunlight exposure.^31^ A review by Sharun et al. found lower infection and mortality rates in countries with greater sunlight intensity and exposure, where there is less risk of vitamin D deficiency.^32^ It was also found that infectious droplets causing surface spread of COVID-19 could be neutralised promptly by ultraviolet, infrared and visible light, with ultraviolet C rays having the fastest action. Our findings are therefore in keeping with prior literature. The relationship between UV exposure, vitamin D levels and COVID-19 outcomes needs further investigation.

## Limitations

There are several limitations to our study. Inherent to our study design, the causality of findings cannot be proved but are only hypothesis-generating. Although the true incidence cannot be determined, this study is an efficient and cost-effective method of estimating prevalence. Regarding vitamin D status, it is difficult to correct for factors prior to admission that affect vitamin D. Due to the use of convenience sampling, our study group may not be a true representation of the population. The strict national lockdown measures may present a bias that underestimates usual sunlight exposure.

## Future recommendations

Further work in understanding the use of vitamin D to prevent or reduce the severity of COVID-19 infections is needed.

## Conclusion

We found a high prevalence of vitamin D deficiency and insufficiency among patients admitted to hospital with COVID-19 and an increased risk for symptomatic disease in vitamin D deficient patients.
